# Extracellular Vesicles as a New Promising Therapy in HIV Infection

**DOI:** 10.3389/fimmu.2021.811471

**Published:** 2022-01-04

**Authors:** Maria A. Navarrete-Muñoz, Carlos Llorens, José M. Benito, Norma Rallón

**Affiliations:** ^1^ HIV and Viral Hepatitis Research Laboratory, Instituto de Investigación Sanitaria Fundación Jiménez Díaz (IIS-FJD), Universidad Autónoma de Madrid (UAM), Madrid, Spain; ^2^ Hospital Universitario Rey Juan Carlos, Móstoles, Spain; ^3^ Biotechvana, Madrid Scientific Park Foundation, Madrid, Spain

**Keywords:** intercellular communication, extracellular vesicles (EVs), HIV infection, immunopathogenesis, clinical application, EVs as therapeutic agents for HIV, EVs as latency reversal agents

## Abstract

Combination antiretroviral therapy (cART) effectively blocks HIV replication but cannot completely eliminate HIV from the body mainly due to establishment of a viral reservoir. To date, clinical strategies designed to replace cART for life and alternatively to eliminate the HIV reservoir have failed. The reduced expression of viral antigens in the latently infected cells is one of the main reasons behind the failure of the strategies to purge the HIV reservoir. This situation has forced the scientific community to search alternative therapeutic strategies to control HIV infection. In this regard, recent findings have pointed out extracellular vesicles as therapeutic agents with enormous potential to control HIV infection. This review focuses on their role as pro-viral and anti-viral factors, as well as their potential therapeutic applications.

## Introduction

Global HIV statistics indicate that around 37.7 million people are living with HIV infection with 1.5 million people newly infected in 2020 (1). Combination antiretroviral therapy (cART) has successfully decreased the associated mortality and consequently it has improved life expectancy (2). The cART can effectively block viral replication reducing plasma viremia to undetectable levels (3). However, cART is not able to completely restore immunological functions (4) and to reduce immune hyperactivation and persistent chronic systemic inflammation caused by HIV (5) which is associated with higher risk of developing cancer, as well as cardiovascular, metabolic, and bone disorders (6–9).

In addition, the cumulative toxicity of cART regimens remains a concern in people living with HIV (10–13). This toxicity added to the obligation of lifelong treatment prompted the research on different strategies with the aim to replace cART for life. These strategies include: a) the reduction of number of target cells available for the virus by CCR5-deficient bone marrow transplant (14–16); b) cART administration very early after primary (17) or acute (18) HIV infection to achieve post-treatment control after drug interruption; c) immunotherapies to delay HIV reactivation by blocking reactivation events (19); d) therapeutic HIV vaccines generated to boost the magnitude, breadth and functionality of HIV-specific immune response (20, 21). However, these strategies have not achieved the expected success, even for the strategy aimed at reducing available CCR5-cells, which is not feasible for the whole HIV^+^ population, it was only successful in only two exceptional patients (14, 15). Thus, it is necessary to focus research on alternative solutions. In this regard, EVs and their observed role in HIV restriction at multiples levels (22–29) lead to consider the potential application of these vesicles in the treatment of HIV infection in order to replace cART treatment.

Moreover, cART cannot completely eliminate the virus from the body, and viral load rapidly re-emergences after 2-8 weeks of cART interruption (30, 31). Establishment of a viral reservoir, very early after acute infection, has been proposed as the main reason for viral rebound and consequently as the main obstacle for HIV eradication. Different types of HIV reservoir have been described. First, the cellular HIV reservoir, formed by specific cells, in a latent state, with HIV-DNA integrated into their genome (32), being the virus invisible to the action of the immune system and of the cART. Second, the anatomical reservoirs, which are sites where the cART and/or the effector cells of the immune system cannot access allowing HIV replication for long periods of time (33).

Several therapeutic strategies specifically designed to eliminate HIV reservoir have been developed with limited success to date. Within them, the most widely explored strategy is the shock and kill approach (34). The goal of this strategy is to eliminate the HIV reservoir, either by cART or immune system, after reactivating it by using latency reversal agents -LRAs-. Despite of the promising results, this strategy has not achieved the expected results (35, 36). The “shock” phase fails to completely reactivate the reservoir, with only <1% of proviruses being reactivated after maximum *in vitro* activation (37). Moreover, this strategy does not discriminate between replication-competent and defective proviruses, and the response to LRAs is widely variable among patients due to the heterogeneous nature of the cellular and anatomical reservoirs and other features such as virus-integration sites and patient-specific aspects (38). Therefore, the search for strategies that can effectively target the HIV reservoir remain open. In this sense, recent findings have pointed out extracellular vesicles (EVs) as potential therapeutic tools to attack HIV infection given their pivotal role in mediating important cell-to-cell communication mechanisms (39).

In this Review, we focus on the understanding how extracellular vesicles mediate intercellular communication in HIV infection, its role as pro-viral and anti-viral factors and its tremendous potential as therapeutic agents to control HIV infection (blocking HIV infectivity and/or reactivating the HIV reservoir).

## Intercellular Communication Mediated by EVs in HIV Infection

Exosomes are membranous EVs of around 40-100 nm, released by many types of cells into the extracellular environment, found in several biological fluids such as blood, urine, semen and breast milk. Most studies use the term “exosomes” to refer to circulating vesicles. However, circulating vesicles are composed of exosomes and microvesicles, and the isolation techniques used do not allow a complete discrimination between them (40). Tetraspanins such as CD63, CD9, and CD81 are normally used as EVs-specific markers. Other proteins, such as Alix and Tsg101 are involved in their biogenesis. It is important to note that HIV virions and exosomes/microvesicles share many features including biophysical and molecular properties, biogenesis and uptake mechanisms. Indeed, Alix and Tsg101 proteins play important roles in the budding of HIV from the host cell (41).

Similarities found between HIV virions and exosomes/microvesicles lead to the so-called “Trojan Exosome Hypothesis”, first proposed by Gould et al. (42). According to this hypothesis, HIV and other retroviruses will take advantage of the host cell exosomal biogenesis machinery for their own benefit. This hypothesis has implications for the virus-host interactions at several levels. The most obvious implication is that the hijacking by HIV of exosomal biogenesis machinery will lead to alternative ways for virus spreading and infection of new target cells, different from the classic direct budding from the plasma membrane (42). Also, the similarity between exosomes and viral biogenesis implies that several viral products can be incorporated into exosomes as several studies have reported (43–48). Full-length unspliced HIV RNA (43), several miRNAs such as the trans-activating response (TAR) element (44–46) and different viral proteins (47, 48) have all been found carried by exosomes of different origin. This ability of HIV to package its own products into exosomes has relevant implications not only for viral spreading but also for viral pathogenesis. In this regard, several studies point to Nef protein as especially relevant in different EVs-mediated mechanisms of viral pathogenesis. First, Nef can promote HIV infection by reducing the expression of CD4 in exosomes and thus neutralizing the ability of CD4-bearing exosomas to act as decoys (49). Second, HIV-infected macrophages can transfer Nef to B cells altering the virus-specific Ig-class switching in lymphoid follicles (50). Virus-specific T-cell responses are also affected by Nef through its modulating effect on both TCR-signalling and cytoskeleton reorganization in T cells. This effect of Nef is dependent on the hijacking of endosomal traffic of protein tyrosine kinase Lck (essential for TCR signaling) and the GTPase Rac1 (essential for cytoskeleton reorganization) (51). Lastly, Nef carried by exosomes has also been involved in promoting chronic inflammation through its effect on lipid rafts formation (reducing the activation of the GTPase Cdc42 and increasing the activation of NLRP3 inflammasome) (52).

In contrast, EVs may contribute to an anti-viral response by delivering HIV restriction factors to nearby cells or by presenting viral antigens (53, 54). Moreover, cell-to-cell communication by EVs could play an important role in the reactivation of the latent HIV (55–60), something of utmost relevance in the search for strategies aimed at eliminating the reservoir.

## Pro-Viral Effects of EVs in HIV Infection

**Figure 1:**
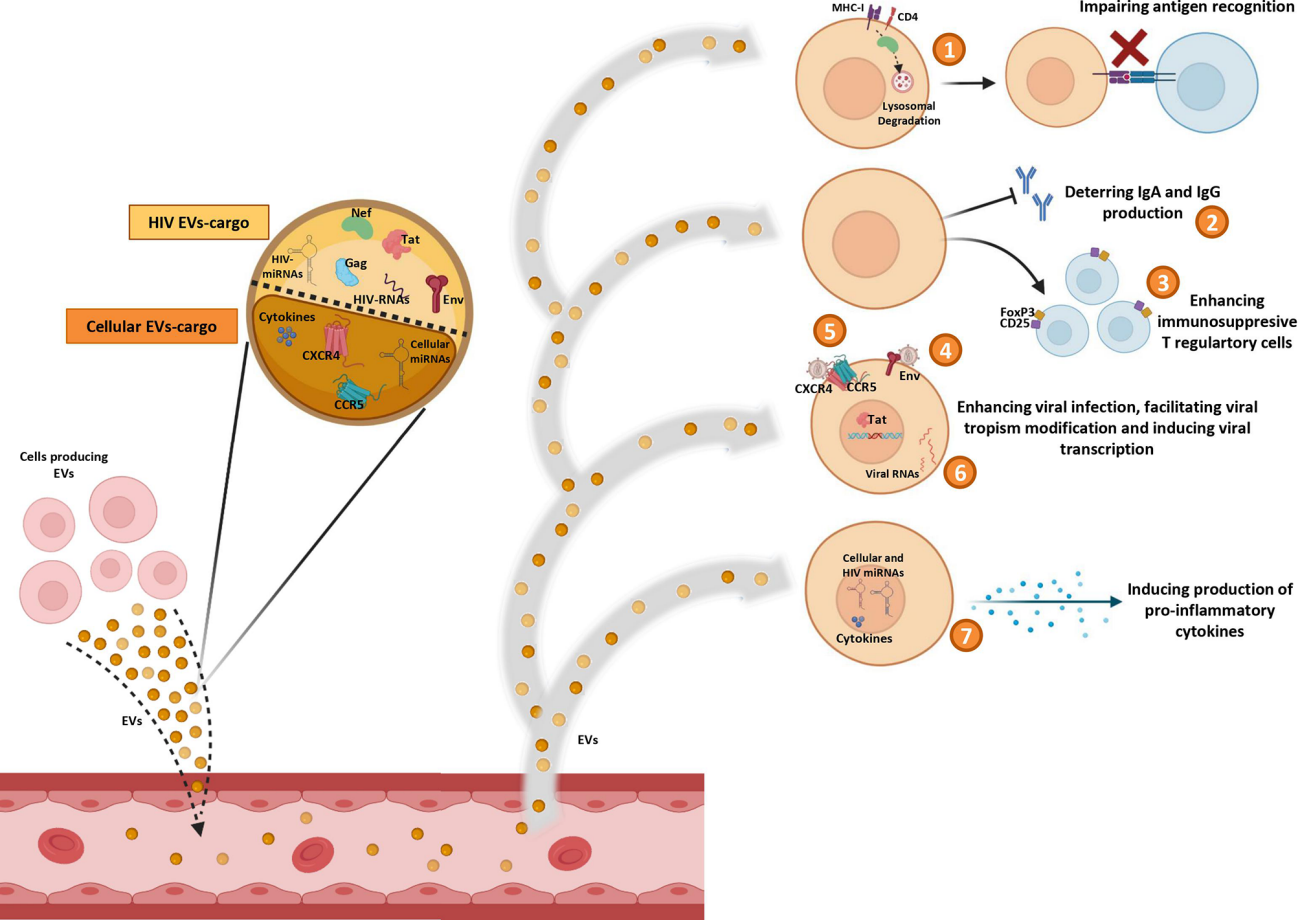
*Pro-HIV actions of factors carried by extracelular vesicles (EVs).* Figure shows Evs produced by different cell sources and released into circulation containing cellular and viral factors that trigger pro-viral effects on target cells: 1) impairing antigen recognition by MHC-I and CD4 lysosomal degradation; 2) deterring IgA and IgG production by B cells; 3) enhancing immunosuppresive T regulatory cells; 4) promoting viral infection by fusing to the target cells with Env protein; 5) facilitating viral tropism modification; 6) activating viral promoter to induce HIV replication; 7) inducing production of pro-inflammatory cytokines.

As has been mentioned, EVs can promote HIV infection (**Figure 1**). HIV-RNA (43), and the HIV proteins Nef (61), Gag, Tat (62), and Env (63) have been observed into EVs from cell culture supernatants. Nef protein can interact with cellular trafficking pathways and induce lysosomal degradation of MHC-I (64, 65) and CD4 (64, 66), disrupting the viral antigen recognition by immune system (67). Moreover, an inhibitory effect of Nef on the adaptive immune response, by deterring the IgA and IgG production in B lymphocytes, has also been described in EVs derived from macrophages of HIV-infected subjects (50). Tat protein acts activating viral promoter to induce HIV replication (62). Gag (62) and Env (63) proteins participate in infection enhancement. EVs released by HIV-infected cells contain Env protein gp120 suggesting that this protein secreted in EVs may promote the virus to attach and fuse to the target cells and facilitate HIV infection (63).

Some HIV-miRNAs have been also observed in EVs from serum of HIV-infected patients and from infected cells culture (44–46). These miRNAs are involved in cytokine production (44–46) and in apoptosis downregulation (44). In general, it seems that EVs can impair the immune response by enhancing immunosuppressive Foxp3^+^CD4^+^CD25^+^ T regulatory cells (68). Furthermore, EVs can act as inducers of the inflammatory state that contributes to HIV disease pathogenesis. Different studies have reported several molecules associated to development of inflammation into EVs, such as: TNFα (69, 70), IFNg (71), IL-12p40, sIL-6R, sTNF-RI, GRO (69), MCP-1, RANTES (72), IL-1α (70, 71), CXCL10 (70), viral Nef protein (that triggers TNF-α release) (73), viral miRNAs (45, 46), and cellular miRNAs (miR-10a-5p, miR-21-5p, miR-27b-3p, miR-122-5p, miR-146a-5p, miR-423-5p) (74). In addition, EVs derived from plasma of HIV subjects increase activation of monocytes/macrophages eliciting the production of inflammatory cytokines (IL-6, IL1-β and TNF-α) by these cells (75).

Interestingly, *in vitro* studies with EVs derived from cell lines culture supernatants (76) and from human peripheral blood mononuclear cells (PBMCs) (76) or platelets (77) of healthy donors, have revealed that virus can be able to use EVs to transfer CCR5 and CXCR4 HIV entry co-receptors to cells in order to facilitate the modification of the viral tropism increasing the number of susceptible target cells (76, 77). However, this ability of EVs to transfer HIV coreceptors between cells could be exploited to combat HIV by engineering exosomes carrying defective correceptors with the aim to prevent HIV transmission, as is the case for the natural delta32-deleted version of host CCR5 (78).

## Anti-Viral Effects of EVs in HIV Infection

Several anti-HIV effects of EVs have been described, such as the presence of MHC-II molecules as part of EVs cargo, a fact that reveals the potential ability of these vesicles to present viral antigens and induce T cells response (54). Tumne et al. revealed that EVs secreted by CD8 + T cells display a potent non-cytotoxic antiretroviral activity that specifically inhibits HIV transcription (79).

**Figure 2:**
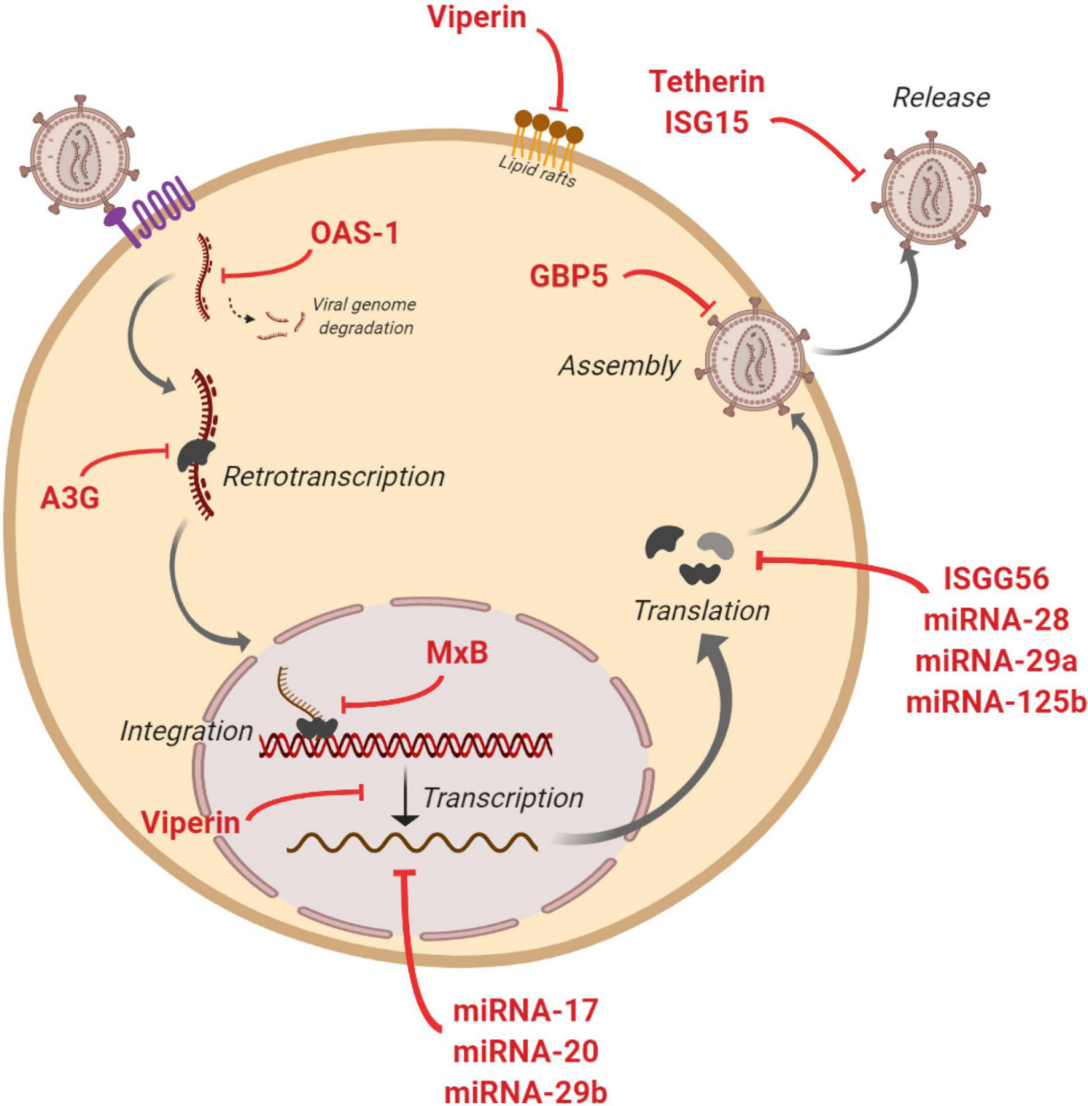
*Anti-HIV actions of factors contained in extracelular vesicles (EVs).* Figure shows inhibitory actions (red lines) at multiples steps of HIV replicative cycle by IFN-stimulated genes and restriction miRNAs carried by EVs.

Some studies have assessed the capacity of EVs to transfer known restriction factors that can inhibit HIV infection in target cells (**Figure 2**). One of these factors is APOBEC3G (A3G - human cytidine deaminase that can cause hypermutation of the viral genome at the retrotranscription step) that has been found into EVs that potently restrict replication of HIV in recipient cells under *in vitro* conditions (80). Moreover, A3G and Tetherin (an interferon-induced protein whose expression blocks the release of HIV), have also been found into EVs derived from human semen (22). At level of mRNA of cellular restriction factors, Tetherin and A3G expression could be induced by EVs. An *in vitro* study showed that EVs from intestinal epithelial cells culture transport antiviral factors at mRNA and protein levels to macrophages, increasing the expression of antiviral IFN-stimulated genes (ISGs) and cellular HIV restriction factors, including Tetherin and A3G, that restricts HIV replication in macrophages (23).

The presence of IFN-stimulated genes ISG15, ISG56, MxB, OAS-1, GBP5, and Viperin with anti-HIV activity has also been reported in EVs (23, 24). These genes code for proteins with diverse functions aimed at blocking HIV: ISG15 protein inhibits virions release; ISG56 restricts viral protein translation; MxB reduces viral DNA integration; OAS-1 activates RNAsa-L to degrade viral genome; GBP5 interferes in the Env protein incorporation to generated virions; and Viperin disturbs lipid rafts and impairs viral replication (**Figure 2**). Moreover, different miRNAs with a protective action against HIV have been found in EVs such as miRNA-17, miRNA-20, miRNA-28, miRNA-29a, miRNA29b and miRNA-125b (23). These anti-HIV miRNAs could regulate HIV expression by directly targeting the virus or by an indirect effect targeting cellular transcription factors. Thus, HIV virus is inhibited at multiple steps of its viral cycle (**Figure 2**). A very recent study has shown that EVs released from human TLR3-activated cervical epithelial cells contain antiviral factors such as multiple IFN-stimulated genes and HIV restriction miRNAs that were able to restrict HIV replication in macrophages in culture. These results suggest that this antiviral mechanism could participate in the innate immunity against HIV infection, and thus a EVs-based delivery system could be considered as a preventive strategy to protect the female reproductive tract against HIV sexual transmission (25).

Concerning the effect of EVs from body fluids, several studies suggest the presence of inhibitory components in semen-derived EVs that can mitigate HIV replication and sexual transmission. EVs derived from semen of healthy donors contain mRNA of Tetherin and A3G that could inhibit HIV infection of various cell types by potentially impairing reverse transcriptase activity (22). Also, these Evs blocked the spread of HIV from vaginal epithelial cells to other cells by restricting cell-to-cell transmission (26). HIV post-entry inhibitory effect of EVs derived from semen of healthy subjects has been proposed by Welch JL et al., in an *in vitro* study in which these EVs blocked HIV proviral transcription by repressing NF-kβ, RNA polymerase II, and Tat recruitment to the LTR region, and thus blocking transcription, initiation, and elongation (27). A recent study enrolling HIV-negative, HIV-infected cART-naïve and HIV-infected cART-treated participants observed that EVs derived from semen inhibited HIV replication *in vitro* independently of donor HIV-infection status (28). In addition, EVs derived from semen of healthy subjects downregulated HIV-induced proinflammatory cytokine production while preserved lymphocyte activation state (81).

EVs from other body fluids have also shown anti-HIV effects. EVs isolated from vaginal fluid could block HIV *in vitro* at post-entry steps, most likely by halving the reverse transcription and the integration processes (29). In EVs derived from breast milk of healthy donors has been shown an *in vitro* protective role. These EVs may bind to monocyte-derived dendritic cells *via* DC-SIGN, a common receptor used by HIV, and consequently, inhibit infection and viral transfer to CD4+ T cells (82).

All this evidence the potential application of these extracellular vesicles in the treatment of HIV infection as a novel and alternative solution to cART for life. EVs can be used as therapeutical agents either by taking advantage of their own natural cargo or by devising ways to engineering their content with different therapeutic agents ranging from anti-HIV drugs to immunomodulating factors. Since EVs function as natural transporters of different molecules between cells and play a pivotal role in intercellular communication, it is expected that they should be the ideal way of delivering different biomolecules to the desired target cells (83). Compared to more traditional approaches of drug delivering including liposome-based or cell-based therapy, EVs offer several advantages such as easier manipulation, long half-life, and higher biocompatibility (84). The feasibility to use EVs as delivery systems has been demonstrated in several studies reporting the use of exosomes as carriers of biomolecules to different types of cells (85, 86). Moreover, recent studies have suggested the potential of EVs as drug delivery systems for treatment of human viral diseases (87, 88). In the setting of HIV infection, the therapeutic potential of EVs is supported by clinical data emerging from other human diseases, especially from the field of cancer (89). Although EVs-based clinical trials for treating HIV have not yet been developed, different approaches have been proposed including the loading of EVs with small molecules with anti-HIV activity (84) and with HIV proteins to generate anti-HIV immune responses (90, 91).

## Role of EVs in HIV Reservoir Reactivation

**Figure 3:**
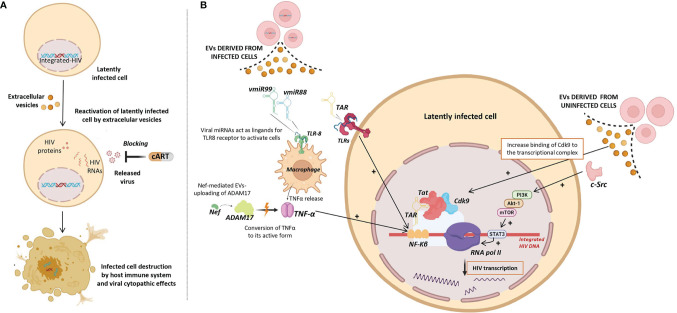
*Role of Extracellular vesicles in HIV reservoir reactivation (EVs).* ***(*A*)*** Potential use of EVs as latency reversing agents (LRAs) to reactivate latently HIV infected cells. Reactivated infected cells produce viral antigens and can be destroyed either by viral cytopathic effects and/or by the host immune system, while released virus can be blocked by combination antiretroviral therapy (cART). **(B)** Different viral factors carried by EVs released from infected cells (left) and EVs released from uninfected cells (right) that could be involved in the induction of HIV transcription and consequently reactivation of HIV latency. Different molecular pathways involved in induction of HIV transcription are shown. Up-regulating actions are represented by black positive arrows.

To date, none of clinical trials using LRAs have reached a significant and persistent reduction of the HIV reservoir. Therefore, an alternative strategy is necessary to efficiently reactivate the transcription of latent HIV as a necessary step to purge the reservoir by the combined action of cART, blocking the released virus, and of the host immune system, destroying productively infected cells (**Figure 3A**). Several studies have found that EVs can reactivate latent HIV infection through different mechanisms (55–60) (**Figure 3B**) as outlined below:

i) Viral transcripts are increased in HIV infected cells when these cells are exposed to purified EVs derived from uninfected cells culture (58). Barclay et al, showed that EVs from uninfected cells culture increased the amount of RNA polymerase II loading onto the HIV promoter, and also increased Cdk9 binding to the transcriptional complex in order to enhance RNA polymerase elongation. In a more recent study, it has been observed that EVs from uninfected cells culture contained activated c-Src (proto-oncogene tyrosine-protein kinase Src) that can trigger the PI3K/AKT-1/mTOR signaling cascade, resulting in the activation of the transcription inducer STAT3, promoting the loading of RNA polymerase II onto the HIV promoter and allowing the reactivation of latent HIV (92). As consequence, larger amounts of viral transcripts were available to be packaged into new EVs and to be exported to uninfected cells.

ii) Tat, a known transactivator of viral transcription, has been detected in EVs isolated from urine of HIV infected patients (93). Tang et al. studied the role of EVs-delivered Tat in the reactivation of viral replication in latently-infected cells (55). For this purpose, the authors engineered human cellular EVs expressing activated Tat protein and they found that EVs-delivered Tat increased the potency of a selected LRA by over 30-fold, measured as change in HIV mRNA expression.

iii) EVs derived from infected cells have also been shown to contain HIV miRNAs that regulate viral and host gene expression (44–46). The most frequent miRNA found in serum EVs of HIV-infected patients is the trans-activation response element (TAR), which down-regulates apoptosis and enhances susceptibility to infection (44). A direct role of TAR in cytokine gene expression has been described by Sampey et al. (46), that observed increased levels of IL-6 and TNF-β in co-cultures of macrophages with EVs derived from HIV *in vitro* infected cells. They suggested that the underlying mechanism by which TAR increases the expression of these cytokines involves the activation of the NF-kβ pathway through the binding of TAR to the TLRs and PKR kinase. Interestingly, NF-kβ regulates the transcriptional activity of the long terminal repeat (LTR) region and therefore, enhances transcription of the HIV genome (94).

iv) Other viral miRNAs such as vmiR88 and vmiR99 have also been observed packaged into EVs derived from serum of HIV-infected patients (45). Bernard et al. found that vmiR88 and vmiR99 could act as ligands for TLR8 signaling that promotes macrophage TNFα release, a cytokine with a pivotal role in the chronic immune activation. TNFα is a pleiotropic protein with several roles in HIV pathogenesis, among them the induction of the nuclear factors binding to the NF-kβ in the LTR, resulting in an increase of HIV RNA expression (95). Interestingly, immature TNFα is converted into its active form by ADAM17, a disintegrin and metalloprotease present in EVs derived from *in vitro* HIV infected cells culture, with a relevant function in the HIV replication in resting CD4+ T cells (60). The packaging of ADAM17 has been observed only in EVs that also contain viral Nef protein. Arenaccio et al. showed that Nef seems to induce the uploading of active ADAM17 in EVs, highlighting the importance of Nef in the mechanism of latent HIV reactivation (60). Reactivation of latent HIV was not observed when Nef was absent or defective (59). Similarly, HIV reactivation was abolished when an inhibitor of the pro-TNFα-processing ADAM17 enzyme or neutralizing antibodies of TNFα were present (59).

All this highlight the relevant role of extracellular vesicles in the reactivation of the latent HIV and its potential application as an innovative and promising strategy aimed at eliminating the reservoir. Some of the studies mentioned above suggest that EVs could be engineered to carry different molecules to act as latency reversing agents. More specifically, the studies of Tan et al. (55), Arenaccio et al. (59) and Barclay et al. (92) clearly demonstrate in *in vitro* systems the ability of EVs to induce HIV transcription and thus reservoir reactivation. These studies are a proof of concept of the potential use of EVs in reservoir reactivation and further studies in the near future are warranted to test the feasibility of this approach in the *in vivo* situation.

## Conclusions and Future Perspectives

The study of EVs in HIV pathogenesis is an emerging field with enormous therapeutic potential to achieve HIV remission. In the current scenario, EVs engineering is possible, with the aim of manipulating their cargo in order to deliver selected molecules to the target cells. Use of EVs for HIV therapeutic purposes may range from modulating immune response, through anti-HIV factors delivery to target cells, in order to block HIV infectivity and control infection, to activating the viral reservoir with the aim to its elimination by either viral cytopathic effects and/or by the host immune system. However, further *in vivo* studies are urgently needed to ascertain the role of EVs in HIV infection and its application at the clinical level.

## Search Strategy and Selection Criteria

Relevant scientific literature was surveyed to review evidence and prepare the manuscript. We searched PubMed for English language papers published until October 2021. Search terms included: “HIV infection”, “Exosomes and HIV”, “Extracellular vesicles and HIV”, “Intercellular communication in HIV infection”, “Exosomes and HIV inhibition”, “Extracellular vesicles and HIV inhibition”, “Proviral effects of Extracellular vesicles in HIV infection”, “Antiviral effects of Extracellular vesicles in HIV infection”, “Toxicity ART and HIV”, “Alternative strategy to ART HIV”, “Strategies HIV functional cure”, “HIV reservoir”, “Exosomes and HIV reservoir”, “Extracellular vesicles and HIV reservoir” and “Role of Extracellular vesicles in HIV reservoir reactivation” Authors screened abstracts for relevance and reviewed full-text articles deemed relevant to the topics address in the manuscript.

## Author Contributions

All authors have participated in the preparation of the manuscript with contributions to draft the manuscript or providing revisions to content. All authors reviewed and approved the final version of the manuscript.

## Funding

This work has been partially funded by the Spanish Directorate General for Research and Technological Comunidad de Madrid fund [grant: IND2018/BMD9651] and the Spanish Carlos III Institute of Health-ISCIII and FEDER fund [grant: PI19/01237]. M-NM is a predoctoral student funded by grant IND2018/BMD9651. NR is supported by the Miguel Servet program funded by the Spanish Health Institute Carlos III [grant: CPII19/00025].

## Conflict of Interest

The authors declare that the research was conducted in the absence of any commercial or financial relationships that could be construed as a potential conflict of interest.

## Publisher’s Note

All claims expressed in this article are solely those of the authors and do not necessarily represent those of their affiliated organizations, or those of the publisher, the editors and the reviewers. Any product that may be evaluated in this article, or claim that may be made by its manufacturer, is not guaranteed or endorsed by the publisher.

## Glossary

**Table:**

HIV	human immunodeficiency virus
cART	combination antiretroviral therapy
DNA	deoxyribonucleic acid
LRAs	latency reversal agents
EVs	extracellular vesicles
CD63	cluster of differentiation 63
CD9	cluster of differentiation 9
CD81	cluster of differentiation 81
Alix	Programmed cell death 6-interacting protein
Tsg101	tumor susceptibility gene 101 protein
RNA	ribonucleic acid
mRNA	messenger RNA
miRNAs	microRNA (small single-stranded non-coding RNA molecule)
Nef	Negative Regulatory Factor
MHC	major histocompatibility complex
CD4	cluster of differentiation 4
Tat	trans-Activator of Transcription
CCR5	C-C chemokine receptor type 5
CXCR4	C-X-C chemokine receptor type 4
PBMCs	peripheral blood mononuclear cells
Env	Envelope glycoprotein
Gp120	Glycoprotein 120
CD8	cluster of differentiation 8
Tregs	regulatory T cells
IgA	Immunoglobulin A
IgG	Immunoglobulin B
APOBEC3G (A3G)	apolipoprotein B mRNA editing enzyme, catalytic polypeptide-like 3G
TLR	Toll-like receptor
ISG	IFN-stimulated genes
ISG15	IFN-stimulated gene 15
ISG56	IFN-stimulated gene 56
MxB	gene coding for myxovirus resistance protein B
OAS-1	gene coding for 2'-5'-oligoadenylate synthetase 1
GBP5	gene coding for guanylate binding protein 5
RNAsa-L	Ribonuclease L
miRNA-17	micro-RNA 17
miRNA-20	micro-RNA 20
miRNA-28	micro-RNA 28
miRNA-29	micro-RNA 29
miRNA-125b	micro-RNA 125b
IFN	Interferon
LTR	long terminal repeat
NF-kβ	nuclear factor kappa-light-chain-enhancer of activated B cells
DC-SIGN	dendritic Cell-Specific Intercellular adhesion molecule-3-Grabbing Non-integrin
Cdk9	Cyclin-dependent kinase 9
c-Src	proto-oncogene tyrosine-protein kinase Src
PI3K	Phosphatidylinositol 3-kinase
AKT-1	RAC-alpha serine/threonine-protein kinase
mTOR	Mechanistic Target of Rapamycin Kinase
STAT3	Signal transducer and activator of transcription 3
TAR	trans-activation response element
IL-6	interleukin-6
TNF-β	tumor necrosis factor beta
PKR	protein kinase R
vimR88	viral micro-RNA 88
vmiR99	viral micro-RNA 99
TLR8	Toll-like receptor 8
TNFα	tumor necrosis factor α
ADAM17	tumor necrosis factor-α-converting enzyme
